# Decision‐making around resuscitation of extremely preterm infants in the Philippines: A consensus guideline

**DOI:** 10.1111/jpc.14552

**Published:** 2019-07-25

**Authors:** Dominic JC Wilkinson, Maria Esterlita Villanueva‐Uy, Dean Hayden, James McTavish, PSNbM Consensus Working Group, Ma. Conchitina T Bandong, Resti Ma. M Bautista, Charito D Corpuz, Luis Emmanuel O Esguerra, Lourdes S Imperial, Jacinto Blas V Mantaring, Socorro de Leon‐Mendoza, Josie Niu‐Kho, Jean S Tay Uyboco, Belen Amparo E Velasco

**Affiliations:** ^1^ Oxford Uehiro Centre for Practical Ethics, Faculty of Philosophy University of Oxford Oxford United Kingdom; ^2^ John Radcliffe Hospital Oxford United Kingdom; ^3^ Murdoch Children's Research Institute Melbourne Victoria Australia; ^4^ Institute of Child Health and Human Development, National Institutes of Health University of the Philippines Manila Philippines; ^5^ Philippine Pediatric Society Quezon City Philippines; ^6^ Philippine Society of Newborn Medicine Quezon City Philippines; ^7^ Verbum Dei Manila Philippines

**Keywords:** clinical decision‐making, consensus development conference, infant, extremely premature, Philippines, practice guideline, resuscitation

## Abstract

While the vast majority of preterm births globally occur in low‐ and middle‐income countries, existing published guidelines relating to the decision‐making and resuscitation of extremely preterm infants (EPIs) largely focus on high‐income countries. In 2018–2019, a working group of the Philippine Society of Newborn Medicine aimed to develop the first national guideline relating to the care of EPIs. The working group reviewed data on the outcomes of EPIs in the Philippines, surveyed paediatricians and neonatologists in the Philippines about current practice and held a consensus workshop. This paper describes the guideline development process and presents a summary of the guidelines. The national guidelines endorse consistency in decision‐making. Health professionals should take into consideration the views and wishes of the infant's parents and the availability of resources to treat the newborn infant. Active management would be appropriate to provide for potentially viable preterm infants at moderate to high risk of poor outcomes, where parents have expressed their wish for this management (and where there are resources available to provide this treatment). For such infants, where parents have expressed their wish to withhold active management, palliative management would also be appropriate to provide. The guideline endorses a grey zone for neonatal resuscitation from approximately 24 to 28 weeks’ gestation in the Philippines, reflecting the context for resuscitation in low‐ and middle‐income countries. Disparities in resource availability are themselves an ethical concern for neonatologists and should be a stimulus for advocacy and improvements in health‐care delivery.

Decision‐making about the care of extremely preterm infants (EPIs) is ethically complex. The most premature infants have a high chance of dying, even with provision of intensive care. To survive, such infants often require a prolonged period of intensive and costly medical treatment, with high rates of serious short‐term complications, including nosocomial infection, intraventricular haemorrhage, bronchopulmonary dysplasia, retinopathy of prematurity and necrotising enterocolitis.^1^, ^2^ In the long term, a proportion of surviving EPIs have severe disability.^3^ Because of the uncertain outcome and significant burden of treatment, resuscitation and intensive care for the most premature infants are regarded as ethically optional, and parents' views about treatment are sought. However, that raises questions for professionals about when this should be applied.

Published guidelines relating to decision‐making and resuscitation of EPIs largely focus on high‐income countries and set aside considerations of limited resources.^4^, ^5^ However, the vast majority of preterm births globally occur in low‐ and middle‐income countries (LMICs), where resource limits can affect the provision of medical care.^6^

Most existing resuscitation guidelines indicate thresholds based on the gestational age (GA) of the infant.^7^ A ‘lower threshold’ marks the GA below which treatment will not usually be provided. An ‘upper threshold’ marks the GA above which treatment is considered mandatory. Between these ages lies a ‘grey zone’, where active treatment may or may not be provided, and parents' wishes are important. In high‐income countries, resuscitation guidelines vary in their details; however, there is considerable overlap in approach. Most such guidelines indicate a lower threshold at 22–23 weeks’ gestation and an upper threshold at 24–25 weeks’ gestation.^5^ There is general agreement that parents' views should be taken into account when making decisions at 23–24 weeks.

An international systematic review of resuscitation guidelines relating to EPIs was unable to identify any guidelines from LMICs.^4^ Studies from LMICs report that a range of different GA thresholds is used by doctors in those settings.^8^, ^9^, ^10^, ^11^, ^12^, ^13^, ^14^, ^15^ As an example, a study from El Salvador reported variable lower thresholds for resuscitation from 25 to 28 weeks.^10^ Practitioners reported a median threshold of 26 weeks for intubation and ventilation and 27 weeks for cardiac massage or pharmacological resuscitation. A study from India indicated that resuscitation would be considered from 28 weeks, while it would usually or always be provided from 32 weeks’ gestation.^16^

The Philippines has a population of 105 million and a GDP *per capita* of US$2989.^17^ World‐wide, it ranks the eighth highest in number of preterm births (350 000 per year).^2^, ^6^ The neonatal mortality rate is estimated to be 14 deaths per 1000 live births, although there are large regional variations (9 neonatal deaths/1000 live births in urban areas, 18/1000 in some rural areas).^18^, ^19^ Child mortality rates in the Philippines are higher than average for East Asia and the Pacific. A total of 1441 hospitals provide for the entire population, of which approximately 67% (966) are privately owned.^20^ As in many other LMICs, for both private and public facilities, parents are often required to pay out of pocket for the care of their infant, although there have been recent government initiatives to provide full funding for newborn treatment for poor families.^21^ In the Philippines, full supportive care and ventilation of a neonate costs approximately US$200 per day in a government hospital. In comparison, the usual monthly wage is only US$300.^22^ Long‐term specialised medical care and allied health support for children with disabilities are unavailable for the majority of the population.

## Development of Guideline

A consensus workshop was held on 6 February 2019 with the aim to produce a guideline to assist clinicians dealing with ethical questions surrounding the care of EPIs. All members of the Philippine Society of Newborn Medicine (PSNbM) were invited to attend the workshop, held prior to the annual conference. The meeting was attended by 95 PSNbM members and representatives of other key stakeholders.

### Outcomes

**Figure 1:**
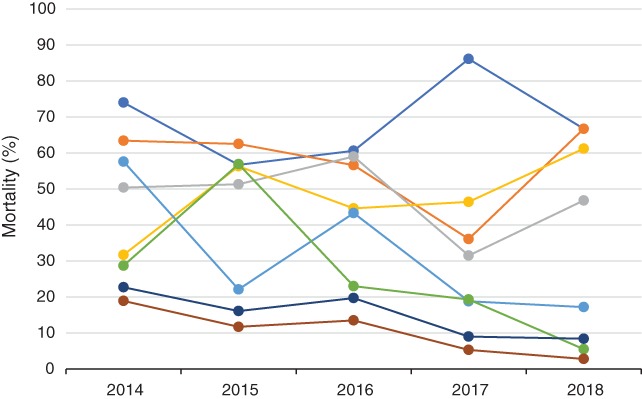
Preterm mortality according to gestational age at the Philippine General Hospital, Manila, 2014–2018. (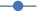), ≤25 weeks; (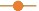), 26 weeks; (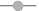), 27 weeks; (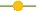), 28 weeks; (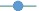), 29 weeks; (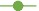), 30 weeks; (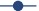), 31 weeks; (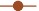), 32 weeks.

The consensus meeting reviewed available outcome data. There is limited published evidence on outcomes for EPIs in the Philippines. Figure 1 illustrates the reported outcome (mortality) for one tertiary hospital in Manila.^23^ There are no available data on long‐term outcomes.

### Existing practice

**Figure 2:**
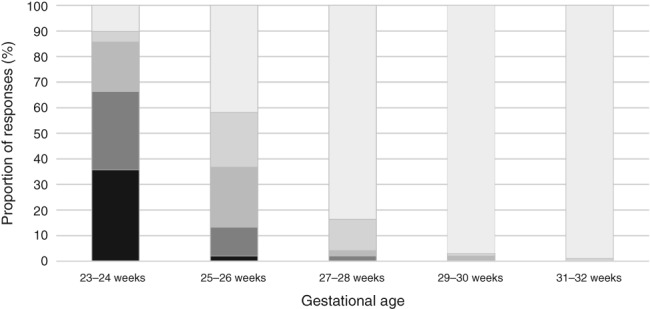
Frequency of initiating resuscitation for a given gestational age as reported in 2018. (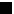), Never; (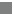), rarely; (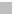), sometimes; (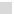), often; (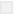), always. (Reproduced from Hayden *et al*.,^24^ with permission).

To aid the development of the consensus guideline, a survey of neonatologists in the Philippines was conducted in 2018.^24^ The survey found that, at 23–24 weeks’ GA, most institutions (66%) would ‘never’ or ‘rarely’ initiate resuscitation, while in a small proportion of hospitals (14%), EPIs would ‘always’ or ‘often’ be resuscitated (Fig. 2). At 25–26 weeks’ gestation, 41% of hospitals would ‘always’ resuscitate, while 21% would ‘often’ and 23% would ‘sometimes’ resuscitate. At 27–28 weeks’ GA, 84% of respondents indicated that they would always resuscitate.

Clinicians' decisions to limit resuscitation were commonly influenced by a desire to respect the parent's wishes but were also influenced by the ‘probability of infant's death’, ‘clinician's morals’, ‘risk of poor quality of life’ and ‘financial cost for family’. There were significant differences between hospitals regarding the resources available (e.g. availability of surfactant, frequency of all ventilators being in use) and the reported outcome for EPIs.

A high proportion of public hospitals (85%) reported that they often or almost always encountered situations where all ventilators are in use and at least one other infant needed ventilatory support. A higher proportion of provincial or district hospitals reported often or always being at maximum ventilator capacity. A total of 40% of public hospitals and 48% of level III/IV hospitals reported that surfactant was only available if parents were able to pay.

In addition to the results of the national survey, ethical considerations, literature surrounding existing guidelines in high‐income settings, current practices in other LMICs and possible guideline models for the Philippine context were presented.

### Development of consensus

A panel discussion involved representatives from Obstetrics, Midwifery, Nursing, Department of Health, UNICEF and the Catholic Church. A statement regarding relevant Christian doctrine and theology was presented by a Catholic priest and bioethicist.

One large interactive group session for all delegates was held. Attendees responded to questions about guidelines for the Philippines via an anonymous live polling tool (Glisser) with the use of their smartphone devices. Consensus was sought in three distinct areas: (i) general principles; (ii) ethical principles; and (iii) specific guidelines.

The results of the consensus meeting are indicated in Box 1. Where relevant, evidence from randomised controlled trials and systematic reviews was used as the basis for practice. However, the majority of elements of the guideline are ethical considerations and therefore based on expert consensus rather than evidence. There was no pre‐specified definition of consensus for the purposes of guideline development. Principles incorporated into guidance were supported by a majority of attendees at the workshop. In considering alternative models for a guideline (e.g. GA‐based, multi‐variable vs. individualised approach), the option with the greatest support by attendees was subsequently included in the guideline.

Box 1 **Results of consensus meeting**

1 Unanimous support for the development of guidelines relating to resuscitation of extremely preterm infants in the Philippines (100% agreement)

2 Strong support for the development of a common set of guidelines that could be applied in all tertiary neonatal intensive care units in the Philippines (81% agreement)

3 Unanimous (or near‐unanimous) support for a set of guiding ethical principles to be the basis for guidance (>90% agreement for each principle)

4 Strong support for the development of an individualised approach to decisions incorporating multiple risk factors into a common framework (68% agreement)

5 Strong support for a framework that accepted the importance of parental wishes in decisions across a wider range of gestational ages (wide grey zone) (73% agreement)

6 Majority support for resuscitation/non‐resuscitation being optional (depending on other factors) between 24 and 28 weeks’ gestation (≥50% agreement)^a^

- Participants varied in the specific gestational age weeks that they would support for the lower or upper thresholds (range from 23 to 27 weeks for lower threshold and 26–28 weeks for the upper threshold). A lower threshold of 24–25 weeks was supported by 62%, while an upper threshold of 28 weeks was supported by 50%.

## Philippine Consensus Guideline (Summarised)

Potential viability in the context of the Philippines refers to extremely preterm birth from 24–28 weeks’ gestation (or 500–1000 g, where GA is uncertain). Ethical principles for the guideline are summarised in Box 2. Where relevant, the level of evidence for certain recommendations is stated.^25^

Box 2 **Ethical principles for the guideline**

Best interests

- In decisions about medical treatment for a very premature infant, the best interests of the child should be the primary consideration.

Family involvement

- The views and values of parents are an important factor in determining whether intensive treatment or comfort care is in the infant's best interest. The child and family should be considered together.

Withholding or withdrawing treatment

- It is ethical to withhold resuscitation from a newborn if that treatment would not be in the best interest of the newborn, would impose an unreasonable burden on the child or family or would constitute an unreasonable use of limited medical resources.
- There is no ethical obligation to provide or continue treatments that are unduly burdensome, extraordinary or disproportionate. Decisions to stop (withdraw) extraordinary treatment are *not* equivalent to euthanasia.

Consideration of financial aspects of care

- Health professionals should aim to provide the best treatment that they can for all newborn infants regardless of the family's ability to pay.
- In considering whether treatment is proportionate, it is ethical to consider the costs of treatment, both for the family and for society. It can be disproportionate and burdensome to provide highly expensive treatment.

Collection of relevant data

- Ethically informed decisions for very premature infants require accurate, up‐to‐date information on the outcome of treatment. There is, therefore, an ethical imperative for those working in newborn care to collect data on the outcomes of patients receiving treatment to inform future decisions.

Comfort care

- Where there has been a decision to stop intensive care because that is no longer considered to be in the best interests of the child and family, there is an ethical imperative to provide high‐quality palliative care (comfort care).

### Antenatal management

Where delivery of a *potentially viable* EPI is anticipated, the obstetric and midwifery team should provide measures to improve the outcome for the premature infant.

#### Antenatal steroids

Consider providing steroids where preterm labour is apparent, GA can be accurately assessed, there is no evidence of maternal infection, active management of the newborn is planned, and facilities are available to provide medical care for the preterm newborn^26^, ^27^ (National Health and Medical Research Council (NHMRC) I).

#### Transfer

If possible and appropriate, arrange timely transfer of the mother to a facility with the ability to provide level III or IV newborn intensive care^28^, ^29^ (NHMRC III). Decisions should take into account the wishes of the parents (including for resuscitation; see below), the safety for the mother and the costs and availability of transfer.

#### Magnesium sulphate

Provide magnesium sulphate where preterm labour is apparent, delivery is imminent and where there is a plan to provide resuscitation of the preterm infant^26^, ^30^ (NHMRC I).

### Antenatal counselling

As early as feasible, where delivery of a potentially viable EPI is anticipated, refer to a neonatologist or paediatrician to enable timely antenatal counselling and decision‐making.

### Decision‐making

Decisions about resuscitation and intensive care for an EPI should take into account an assessment of risk for the infant and the wishes of the parents.

#### Assessment of risk

It is important to assess, for an individual situation, the realistic chance of survival and of severe morbidity if resuscitation and intensive care are attempted. This assessment should include all known risk factors relevant to the infant and the resources available to provide treatment if/when the infant is delivered.

##### Assessment of GA and expected birthweight

Assess the GA of the preterm infant. If gestation is uncertain, assessment of the expected birthweight may help decision‐making.

In the Philippines, infants from 24 to 28 weeks’ gestation (or with an anticipated birthweight of 500–1000 g where GA is uncertain) are potentially viable. These are not absolute cut‐off points.

##### Assessment of modifiable risk factors

**Figure 3:**
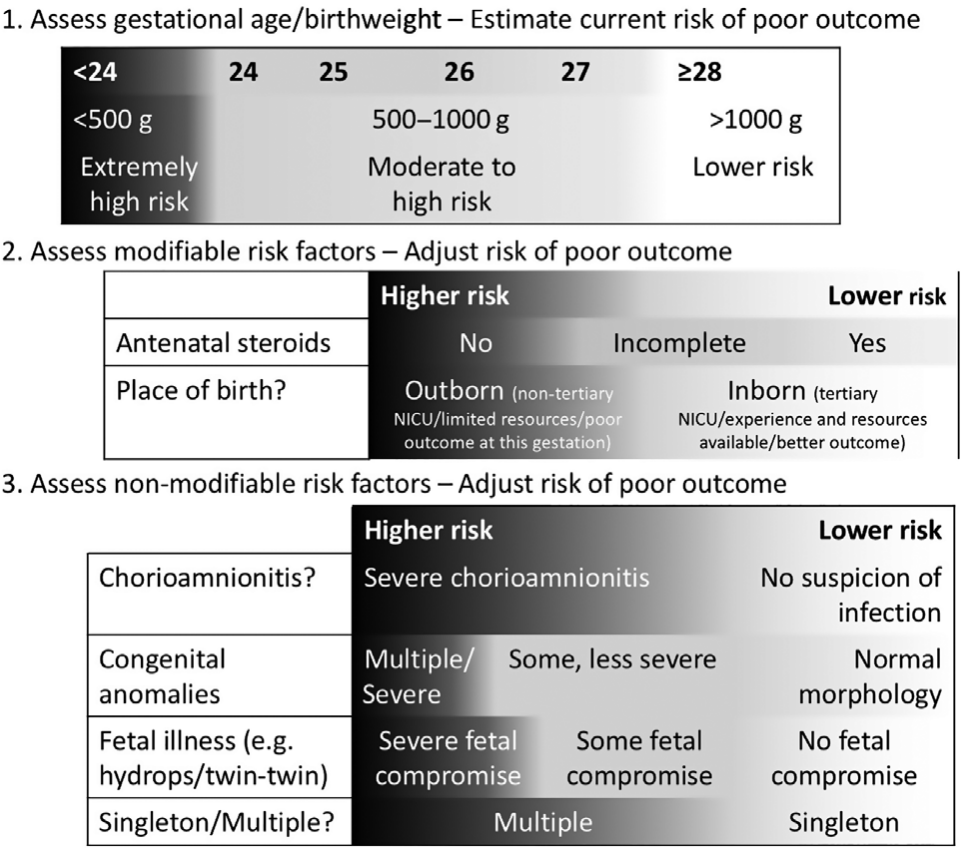
Multi‐variable model for the assessment of risk for the extremely preterm infant in the Philippines. NICU, neonatal intensive care unit.

Consider potentially modifiable factors that may influence the outcome of the EPI (Fig. 3). Clinicians should consider local experience to assess the realistic chance of survival or severe morbidity of the infant. Wherever possible, this should be based on relevant, accurate and recent local data.

Where possible and appropriate, these risk factors should be modified through the provision of antenatal steroids and/or transfer. If this occurs, the risk to the infant should then be re‐evaluated.

##### Assessment of non‐modifiable risk factors

Consider additional risk factors that may influence the outcome of the preterm infant, for example, evidence of severe chorioamnionitis, severe or multiple congenital anomalies, fetal hydrops, multiple pregnancy and twin‐to‐twin transfusion.

The modifiable and non‐modifiable risk factors will influence the risk and expected outcome of an individual infant. For example, the presence of adverse risk factors (e.g. severe fetal illness or anticipated delivery in a centre with limited resources or poor outcome at this gestation) may mean that a potentially viable infant has an extremely high risk of dying or severe morbidity if resuscitation and intensive care are attempted. The absence of adverse risk factors or greater experience/resources at the local centre may imply a lower risk of dying or severe morbidity for a potentially viable infant.

#### Assessment of parental wishes

The estimated chance of survival, as well as the risks of morbidity, of the EPI should be sensitively but realistically conveyed to the parents. Parents should be provided with information about the nature and burden of treatment required, including the local experience with caring for infants at the expected gestation. If applicable, parents should be provided with realistic estimates of the costs that they will potentially bear for the treatment. This should also include information about assistance or external support available for those costs.

Health professionals should ascertain parents' desires about resuscitation and initiation of intensive care if preterm delivery ensues.

#### Resuscitation decision

Following the assessment of risk and of parental wishes, a decision should be made to provide either active neonatal management or comfort care.

##### Active neonatal management

For EPIs at lower risk of poor outcome, it would be appropriate to attempt resuscitation at delivery and then admit the EPIs to the neonatal intensive care unit. For example, this would apply to most infants ≥28 weeks’ gestation.

Active management would also be appropriate for potentially viable preterm infants at moderate to high risk of poor outcome, where parents have expressed their wish for this management (and where there are resources available to provide this treatment).

Active neonatal management for EPIs includes thermal management, airway management and respiratory support.^31^

##### Palliative neonatal management

For EPIs at extremely high risk of poor outcome (i.e. dying or severe morbidity), it would be appropriate to provide palliative management (comfort care) at delivery. For example, this would apply to most infants born <24 weeks’ gestation.

Palliative management would also be appropriate to provide for potentially viable infants at moderate to high risk of poor outcome, where parents have expressed their wish to withhold active management.

Palliative neonatal management includes avoidance of interventions that would not be in the infant's best interests, as well as the provision of measures focused on the infant's comfort and holistic care of the needs of the family.

##### Situations of uncertainty

In situations where there is uncertainty about the risk to an infant or uncertainty about parental wishes, a provisional plan for active neonatal management should be pursued.

Note that the assessment of gestation and of viability at the time of delivery is not necessarily reliable.^32^

### Subsequent decisions

As with all medical treatment, decisions to initiate active management for an EPI should be reviewed and reconsidered if additional information comes to light or if circumstances change. For example, following admission to the neonatal intensive care unit, further information may become available about the infant's chance of survival or of severe morbidity (such as a lethal congenital anomaly or severe interventricular haemorrhage). Alternatively, complications of treatment may develop (e.g. severe nosocomial infection). There should be continuous discussions with parents about the infant's progress and outlook.

Where it becomes apparent that an EPI has a very low chance of survival if treatment continues, has a high chance of severe morbidity or requires treatment that would be extremely burdensome for the infant or for the family, there is no ethical obligation to continue treatment. In such circumstances, the medical team should discuss the infant's condition with the parents and consider withholding or withdrawing active treatment and providing palliative care.

Where there is a plan to move to palliative care, arrange for privacy for the family as much as possible. Treatments should be reviewed – those aimed at survival or recovery should be discontinued. Removal of endotracheal tubes or other devices is a medical responsibility; the family should not be required to perform this procedure.

## Conclusions

This paper has described the development of a national guideline relating to decision‐making and resuscitation of EPIs in the Philippines.

There are several differences between this guideline and those published in high‐income countries. The grey zone for neonatal resuscitation in the guideline is higher and wider than in other international guidelines. This reflects different outcomes for EPIs in the Philippines but also the significance of limited resources and the discrepancies in resources and outcomes between different parts of the health system. The document explicitly discusses issues relating to the costs of treatment borne by families and acknowledges that these costs are an ethically relevant consideration for families. However, the document also recognises that disparities in resource availability and outcome are an ethical concern for neonatologists and should be a stimulus for advocacy and improvements in health‐care delivery.

To our knowledge, this is the first national guidance on resuscitation decisions for EPIs in an LMIC. The Philippine document may prove a useful model for health professionals in other resource‐limited settings.
